# How can information systems provide support to nurses’ hand hygiene performance? Using gamification and indoor location to improve hand hygiene awareness and reduce hospital infections

**DOI:** 10.1186/s12911-017-0410-z

**Published:** 2017-01-31

**Authors:** Rita Marques, João Gregório, Fernando Pinheiro, Pedro Póvoa, Miguel Mira da Silva, Luís Velez Lapão

**Affiliations:** 1 Global Health and Tropical Medicine, Instituto de Higiene e Medicina Tropical, Universidade Nova de Lisboa, Rua da Junqueira 100, 1349-008 Lisboa, Portugal; 2 Instituto Superior Técnico — Universidade de Lisboa, Avenida Rovisco Pais 682, 1049-001 Lisboa, Portugal; 3 Centro Hospitalar de Lisboa Ocidental — Hospital S. Francisco Xavier, Estrada do Forte do Alto do Duque, 1449-005 Lisboa, Portugal; 4 NOVA Medical School, CEDOC, Universidade Nova de Lisboa, Campo Mártires da Pátria 130, 1169-056 Lisboa, Portugal

**Keywords:** Design science research, Hospital-acquired infections, Hand hygiene compliance, Automated monitoring system, Gamification, Behaviour changing

## Abstract

**Background:**

Hospital-acquired infections are still amongst the major problems health systems are facing. Their occurrence can lead to higher morbidity and mortality rates, increased length of hospital stay, and higher costs for both hospital and patients. Performing hand hygiene is a simple and inexpensive prevention measure, but healthcare workers’ compliance with it is often far from ideal. To raise awareness regarding hand hygiene compliance, individual behaviour change and performance optimization, we aimed to develop a gamification solution that collects data and provides real-time feedback accurately in a fun and engaging way.

**Methods:**

A Design Science Research Methodology (DSRM) was used to conduct this work. DSRM is useful to study the link between research and professional practices by designing, implementing and evaluating artifacts that address a specific need. It follows a development cycle (or iteration) composed by six activities. Two work iterations were performed applying gamification components, each using a different indoor location technology. Preliminary experiments, simulations and field studies were performed in an Intensive Care Unit (ICU) of a Portuguese tertiary hospital. Nurses working on this ICU were in a focus group during the research, participating in several sessions across the implementation process.

**Results:**

Nurses enjoyed the concept and considered that it allows for a unique opportunity to receive feedback regarding their performance. Tests performed on the indoor location technology applied in the first iteration regarding distances estimation presented an unacceptable lack of accuracy. Using a proximity-based technique, it was possible to identify the sequence of positions, but beacons presented an unstable behaviour. In the second work iteration, a different indoor location technology was explored but it did not work properly, so there was no chance of testing the solution as a whole (gamification application included).

**Conclusions:**

Combining automated monitoring systems with gamification seems to be an innovative and promising approach, based on the already achieved results. Involving nurses in the project since the beginning allowed to align the solution with their needs. Despite strong evolution through recent years, indoor location technologies are still not ready to be applied in the healthcare field with nursing wards.

**Electronic supplementary material:**

The online version of this article (doi:10.1186/s12911-017-0410-z) contains supplementary material, which is available to authorized users.

## Background

Hospital-acquired infections (HAIs) are infections acquired by patients in a hospital facility while receiving treatment for medical or surgical conditions, and in whom the infection was not present nor in incubation at the time of admission [1, 2]. Although partially preventable, these infections cause more deaths than AIDS, breast cancer and car accidents altogether [3]. It is still one of the biggest preventable problems healthcare is facing, leading directly to around 37 000 deaths, 16 million extra-days of hospital stay and €7 billion financial losses of direct costs, only in the Europe [1]. Among the several factors responsible for infections in a healthcare setting, the study of healthcare workers (HCWs)’s hand hygiene (HH) compliance has gained importance in the last decades.

HH is an action of hand cleansing [4], and can refer to either:

- **Hand wash:** washing hands with plain or antimicrobial soap, which should be done when hands are visibly dirty or soiled, after using the toilet or when alcohol-based hand rub (ABHR) is not available. It must last between 40 and 60 s.
- **Hand antisepsis:** application of antiseptic hand rub or performing antiseptic hand wash as routine HH. It must last between 20 and 30 s.
- **Surgical hand scrub:** application of antiseptic hand rub or performing antiseptic hand wash by a surgical team before an operation. It must last more than 5 min.

Performing HH at the right times is a simple and inexpensive prevention measure that can save lives [5], but compliance is still suboptimal amongst HCWs [6]. Chatterjee et al. studied HCWs’ HH compliance rate in an ICU of a tertiary care hospital in India. During 3 months, 20-min direct observation sessions were performed. Overall compliance of the study was approximately 11,96% [7], which is dangerously low. Factors like forgetfulness, lack of time, lack of guidelines’ knowledge, have been declared as barriers to better compliance [4]. Therefore, it becomes crucial to understand and investigate ways to improve HH compliance.

Monitoring HCW’s HH performance is an important element of multimodal HH promotion programs [8]. To assist this, World Health Organization (WHO) proposed a framework for understanding, training, observing and communicating HH performance [4]. The “My five moments for hand hygiene” framework links specific moments to HH opportunities, and it is further detailed in Additional file 1 Appendix I. This framework provides information that can be used to calculate the HH compliance rate, given by the reason between the number of complied moments and the total number of occurred moments.

Direct observation of HCWs’ HH practice by professional observers, is the standard for monitoring HH compliance. Despite being the method that provides the most accurate data and the only one that allows technique evaluation, it is costly, time-consuming and susceptible to several biases like the Hawthorne effect.

Hospitals need to come up with innovative ways of monitoring HCWs’ HH performance and provide them with proper feedback. McInnes et al. conducted a qualitative study that aimed at understanding the perspectives of senior managers regarding current and future strategies to improve HH compliance [9]. Alongside several interesting issues mentioned, the need of having individualized and immediate real-time feedback of HH practices must be highlighted. In this case participants complained that no meaningful information could be extracted from the aggregated audit results, which often are disseminated long time after being collected.

Automated monitoring systems can help providing this timely and personalized feedback. These systems can electronically identify when an HCW uses a sink or an ABHR dispenser and generate exact quantitative results from automatically collected data [4], which can be used to examine trends regarding HH compliance value over time. These systems cope with direct observation method’s main limitation, which is the impossibility to collect continuous data during a period of time. However, there are some remaining questions to concern about: accuracy, costs (which can be high, depending on the chosen technology), ethical questions (HCWs can be sceptical about being monitored this way), etc. Some studies, like those performed by Levchenko et al. [10] and Swoboda et al. [11], are attempting to prove that these solutions can effectively lead to a better HH compliance, and so far they appear to be promising in improving monitoring performance and HH compliance among HCW. For example, Møller-Sørensen and colleagues tested a dispensing technology that reminds HCWs, patients and visitors to perform HH after restroom visits. Despite its limitations, this system was able to improve HH compliance to around 90% [12]. In fact, automated monitoring systems are not the only successful real-time solutions developed in the context of healthcare. The real-time surveillance system to monitor HAIs, proposed by Du et al. [13], and the diagnostic reminder system that assists clinicians’ decision-making, proposed by Ramnaraya et al [14], are two good examples.

Some automated monitoring systems are based on real-time location. For those, one important component is the indoor positioning system (IPS), which continuously and in real-time determines the location of a target in an indoor space [15].

Gamification is a recent but sought-after approach which can be defined as “the use of game elements and game-design in non-game contexts” [16] to “engage and motivate people to achieve their goals” [17], providing a whole different user experience. It aims at stimulating people’s intrinsic motivation in doing an activity, related to a real-world problem and goal, by trying to make it rewarding for itself. In this way, gamification creates incentives without incurring into high costs. Despite being related to gaming, gamified systems are not full-fledge; they just use parts of parts of games in an already existing process [18].

Based on the evidence that automated monitoring systems are capable of improving HH compliance, we can augment their capabilities by using the potential of games to leverage HCWs motivation in performing HH, thus increasing HH compliance. In fact, there is some evidence that gamification and game-like initiatives can improve compliance with clinical procedures. McKewon and colleagues launched the “150 Lives in 150 Days” campaign in British Columbia, aiming at increasing engagement with sepsis care protocols. To achieve this, a gamification approach was used, applying game elements like leaderboards and feedback. Its high adherence resulted in increasing protocol awareness and compliance, and in one region of the province severe sepsis mortality rate decreased to one of the best results [19]. Greenly presented “Helping Hippocrates”, a game involving staff and patients to ensure compliance with the procedure of checking patients’ wristbands. A patient was selected to wear a band with the name Hippocrates, and HCWs had to check all wristbands to find Hippocrates. Following this process, other wristbands’ information would be checked, which was the goal of the project. This initiative resulted in a decline from 8.2% to a sustained zero in patient identification errors [20].

To address the problem of the poor compliance of HCWs to HH, the aim of this study was to develop a gamification solution that can provide HCWs’ real time feedback on personal HH compliance. The goal is to create awareness regarding HCWs’ HH compliance, while trying to change their behaviours and optimize their performance.

## Methods

### Methodology overview

To accomplish the proposed objective, Design Science Research Methodology (DSRM) was adopted as the methodology to conduct this research, because it is based on an iterative process. This allows to incrementally design, develop, test and evaluate a solution that is aligned with the organization and end users’ needs [21].

Hevner et al. [22] have established guidelines for a DSRM project. These guidelines incorporate six activities as suggested by Peffers et al. [21], each with a specific set of tasks to be carried out with an appropriate method (Table 1).

**Table 1:** Design Science Research activities and tasks to perform

DSRM Activity	Method/Tasks
1. Diagnose of current situation and identification of problem relevance	Survey and observational time-and-motion study
2. Defining the objectives for a solution	HH moments and nurses ward behaviour analysis
3. Design and development	Design of the artifact
4. Demonstration	Preliminary experiments, simulations, field studies, focus groups
5. Evaluation	Analysis of activity 4’s results and focus groups
6. Communication	Done throughout the duration of the project, through journal or conference papers such as this.

The focus of the current paper is to present the methods and results from activities 3, 4 and 5. This paper itself is part of activity 6. The outcomes from the remaining activities are to be presented elsewhere.

The main output of a DSRM project must be an artifact. This is initially developed in activity 3 and is then demonstrated and evaluated iteratively in order to obtain information that allows its continuous improvement in next iterations’ activity 3.

Three artifacts were developed under the scope of this work: two models and one instantiation.

### Problem identification and goals for a solution

The starting point for the Information System (IS) design was the recognition of HCWs’ dissatisfactory HH practices as a subject of human behaviour [23]. Therefore, the solution should promote a change of these behaviours in order to produce the desired outcomes. This combined with HCWs evident need of having individualized and timely feedback regarding their HH practices lead to the idea of creating a decision-support system that provides real-time HH feedback. To ensure that the solution would be different from the already existent automated monitoring systems, gamification was selected as the main feature. It was considered that gamification would allow to explore and work on the human factors that leverage motivation and encourage behaviour change.

### Design and development

**Fig. 1:**
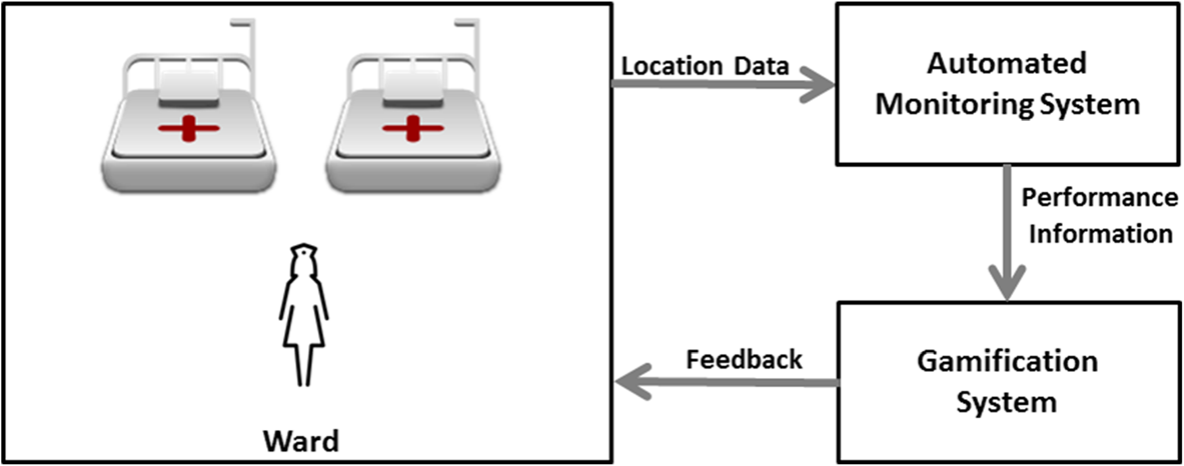
IS abstract model

The IS model is represented in Fig. 1. Without interfering with HCWs’ regular practices, real-time data regarding HH opportunities and actions is automatically and continuously recorded using an automatic monitoring system. The IS is intended to function in non-surgical settings, thus it will only focus on hand wash and hand antisepsis actions, measuring their duration as a proxy to HH compliance. Also, compliance assessment was solely based on time, while technique was not evaluated. To achieve this, HCWs’ proximity to strategic locations and respective timestamps are first collected using an IPS. Locations where HH actions take place are validated in a special way, accordingly to HCW’s proximity duration. Proximity to a sink (where hand wash is performed) was registered after 40 s near this facility, while proximity to ABHR dispensers (where the product to perform hand antisepsis was acquired) was registered after 4 s, taking into consideration that the HCW can perform the action away from the dispenser. Other locations (like beds) were registered by the time they are detected. Since sinks and ABHR dispensers are isolated from other interest areas (such as zones for medicine preparation or direct patient care), this technique can be implemented. For example, one could argue that a location at a bottom of a bed could have a reason other than extracting ABHR product to perform HH. However, since the patient-care always takes place near a patient, it is not likely that a HCW would stand there for that reason.

Afterwards, location data was then analysed with an algorithm, which implements the business rules that state when HH compliance occurs (for example: “If the HCW gets close to the patient’s bed, he/she shall perform HH”).

**Fig. 2:**
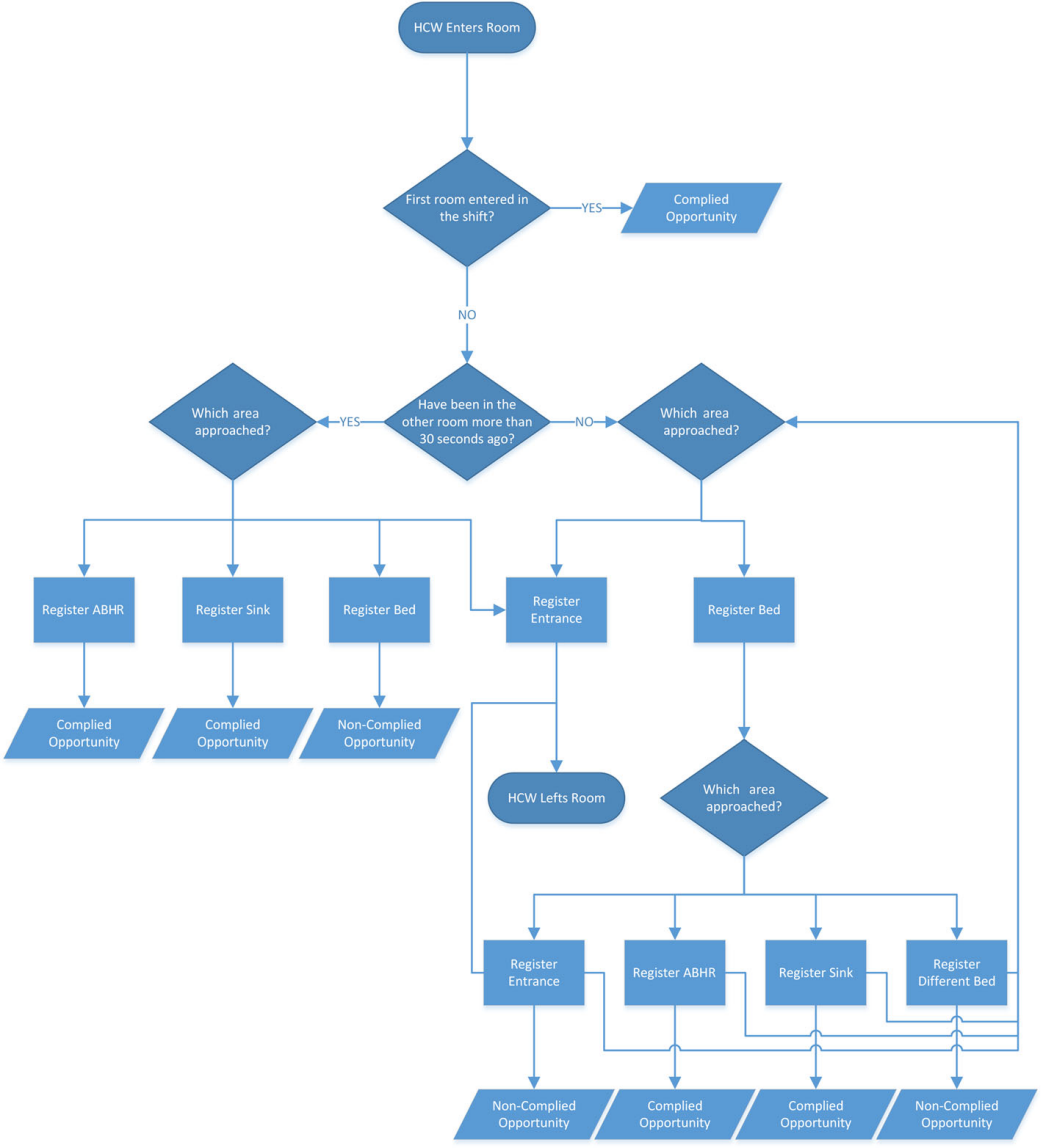
Model of the business rules that state when HH compliance occurs

These rules were structured in a second model artifact, which is presented in Fig. 2. It represents the different HH opportunities that may arise and how they are compiled or not, following WHO’s “My five moments for hand hygiene” framework.

Finally, this information is presented to the HCWs using a gamification solution that provides feedback information in real-time. The building blocks used to create this gamified system were selected from the list of game elements proposed by Werbach and Hunter [16], which is presented and discussed in Additional file 2: Appendix II. Those elements were considered to be the ones that better serve the research propose. The third artifact is an instantiation that implements the two presented models. Because it has changed through the two work iterations performed, concrete implementation details are to be presented in the subsections that close this section, each corresponding to one iteration. Nevertheless, attention was paid in designing a tool such that the additional workload is as negligible as possible, since this can represent a very impeditive barrier to the effectiveness of the project [24].

### Demonstration

After the initial design was settled, the demonstration activity could begin. The objective of this activity is to demonstrate that our artifact can be used to solve the stated problem, for which purpose a range of methods was selected. The aim of the demonstration was to test the implementation in two phases with the same duration. First, it would only monitor HCWs movements in the ward, without using the gamification solution, and then the gamification solution was made available. This would allow to compare HCWs’ behaviour with and without the influence of the gamification solution.

One of these methods was focus groups with end users, which were performed to collect feedback from and involve end users in the process since the beginning. These focus groups were conducted as unstructured interviews (meaning that no questions were established a priori), where the system was presented and participants would contribute by sharing their feelings and opinions regarding it. Thoughts were registered, and main conclusions were written down, decided by agreement between researchers and participants. It was necessary to collect written consents from participants, stating what data was to be collected, why and where along with the final use. The focus group with the participating nurses allowed to get information in order to improve the initial instantiation and prepare it for the following one.

Further demonstration activities are presented in the following subsections, each corresponding to one work iteration.

### First iteration

For the first iteration, it was decided to build an IPS from scratch. To accomplish this Estimote® beacons technology were used [25]. Estimote® beacons are commercial wireless sensors which broadcast Bluetooth Low Energy (BLE) signals that applications, installed on mobile devices, receive and interpret in order to trigger some task, depending on the source beacon (like displaying a notification). A Software Development Kit (to easily create applications) and a Demo app (to perform some tests like estimating proximity) are included in the product. Estimote® beacons were chosen since they were the least expensive option and promised accurate values using a proximity-based technique.

### Preliminary tests

**Fig. 3:**
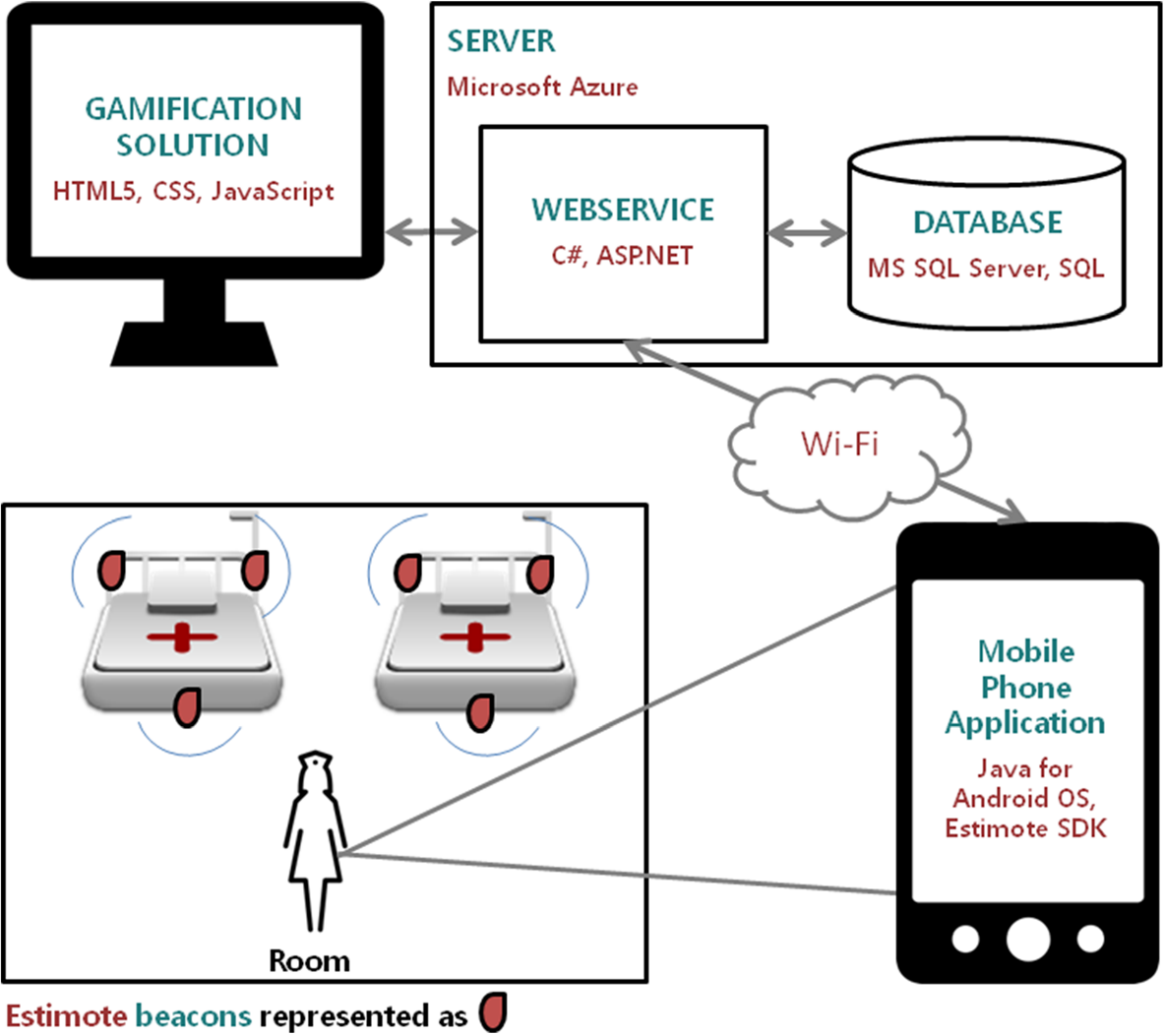
Technological architecture of the first instantiation

Before starting the development of the first instantiation, some preliminary and informal tests were conducted on a pack of three Estimote® beacons to check whether or not the technology fitted the project’s needs. After these tests, the development of this instantiation, whose technological architecture is presented in Fig. 3, could begin.

### Design and development of first instantiation — automated monitoring system component

Following this architecture, beacons were to be placed in strategic places in the room (to register the operational actions of nurses): one at entrance, two near the beds, one per ABHR container and one per sink. These equipments used BLE technology to send signals, which were detected by an Android application installed on a mobile phone carried by the HCW in his/her pocket. Using these signals, the application was able to detect if it is near the beacon or not. For example, if the application detects strong signals from a beacon placed in a sink, then it will know that the HCW is near that sink. This information was continuously processed using the already mentioned algorithm (which implements the model from Fig. 2), and every time an HH moment was detected and validated (or not), the application sent this information to a web service, which in turn updated the database.

**Fig. 4:**
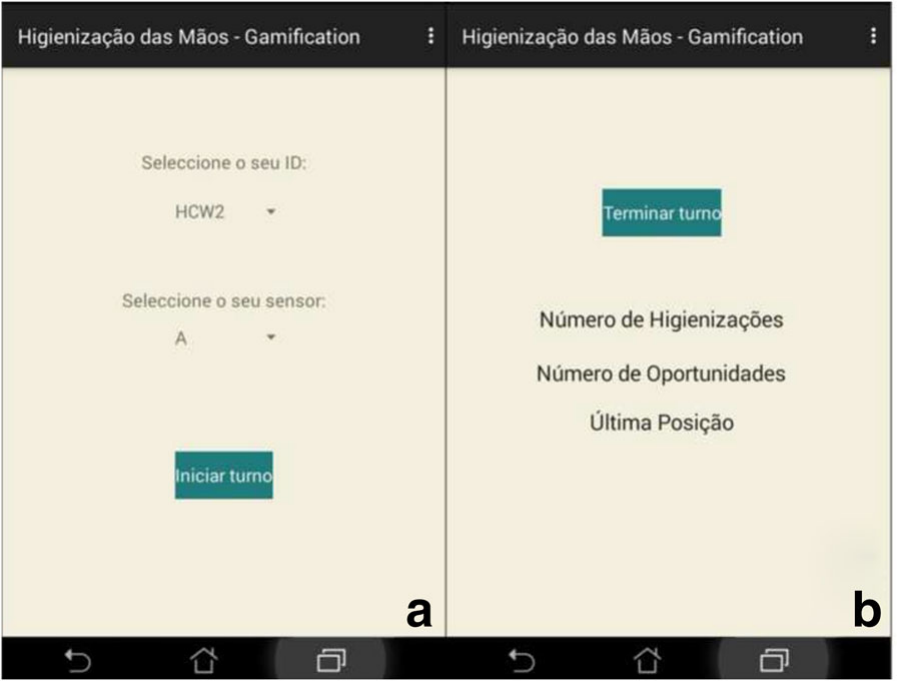
Android App. **a** A screen for the HCW to select his/her ID and the ID of the sensor (s)he will be using. After clicking on the button, (s)he can start the shift; **b** The button allows a HCW to end the shift, thus the monitoring. Information is presented regarding the number of HH actions, number of HH opportunities and the last position where the HCW was detected, respectively

HCWs interact with the application only to mark the start and end of a shift. To start it, a HCW picks a smartphone in the hospital and selects his/her name (or a colour) in the application. During the shift, the application continuously presents information regarding the number of opportunities, how many of them were compliant and the last position where HCW’s presence was detected. At the end of the shift, the HCW only has to click a specific button to end it for the day. In Fig. 4 snapshots of these two screens are presented.

### Design and development of first instantiation — gamification component

**Fig. 5:**
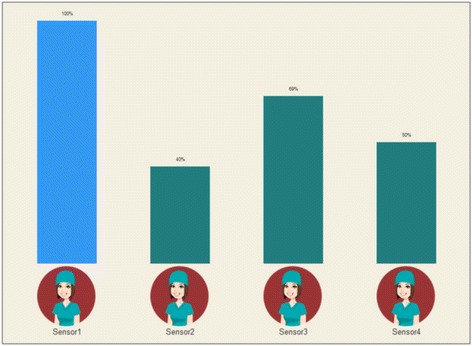
Dashboard component of the first instantiation of the gamification solution

Regarding the gamification solution, a dashboard screen, where HCWs’ HH compliance information was displayed during the shift, was developed, providing real-time feedback to nurses via a monitor placed in the nurses’ room (Fig. 5).

This solution presents a bar chart composed by several bars (corresponding to the number of HCWs working per shift) indicating HH compliance performance value (the “shift score”). Each bar was illustrated with similar avatars, just to provide an element of entertainment. HCWs’ real names were not presented in the dashboard in order to protect their privacy. Therefore, a HCW will only be able to know which sensor he/she was using, but will not know which sensors (e.g. the avatar’s number) its colleagues were using.

By using these elements, feedback (provided regarding HH performance), competition (implemented by presenting the currently highest score with a bar of different color) and win state (the player with the highest score at the end of the shift was the winner) mechanics were explored.

### Simulation of the first instantiation

Subsequently, a simulation was conducted to fully test the instantiation.

### Second iteration

**Fig. 6:**
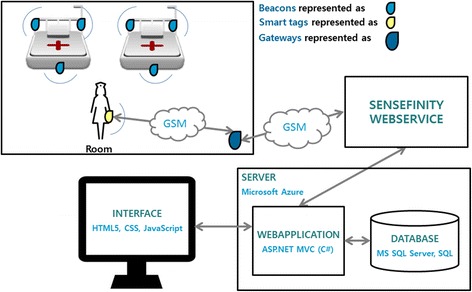
Technological architecture of the second instantiation

As further described in the Results section, accuracy provided by Estimote® beacons was not enough to cope with the system’s demands. This required the team to develop a second instantiationusing a different indoor location technology. The technological architecture of the second instantiation, presented in Fig. 6, was very similar to the initial instantiation.

For the IPS, a partnership with Sensefinity® was initiated [26]. Sensefinity® is a Portuguese start-up focused on the development of disruptive solutions related to the “Internet of Things”. Among their products, they are developing smart beacons that use both Bluetooth and a proprietary protocol (also operating on the 2.4 GHz frequency band) to communicate. By combining this technology with other ones (like smart tags and gateways), Sensefinity® creates location products that are aligned with their clients’ needs.

### Design and development of second instantiation — automated monitoring system component

As in the first instantiation, the system was built using a proximity based technique. Beacons emit signals that tags, which substitute mobile phones from the previous instantiation, can detect. Whenever tags are approaching or walking away from a beacon, they send a message composed by its identification, detected beacon identification, current type and type of message (approaching or leaving) to gateways, which in turn send these messages to a server using Global System for Mobile Communications (GSM) technology. This means that, ideally, the IPS should register one (and only one) message each time a tag enters a beacon range, and one (and only one) message each time a tag exits a beacon range. Potential interferences can make these values variate, so it is important to have algorithms analysing and filtering these messages. By doing this, HCWs’ positions over time can be detected. Beacons were to be placed in the same position, but only one beacon (with a wider range) per bed and a beacon in a central point of the room, instead of being at the entrance.

Due to the fact that in this version of the instantiation there was no mobile phones usage, HCWs had to indicate the moments when they started and ended the shift using a different method. This logic was applied in the dashboard screen.

### Design and development of second instantiation — gamification component

**Fig. 7:**
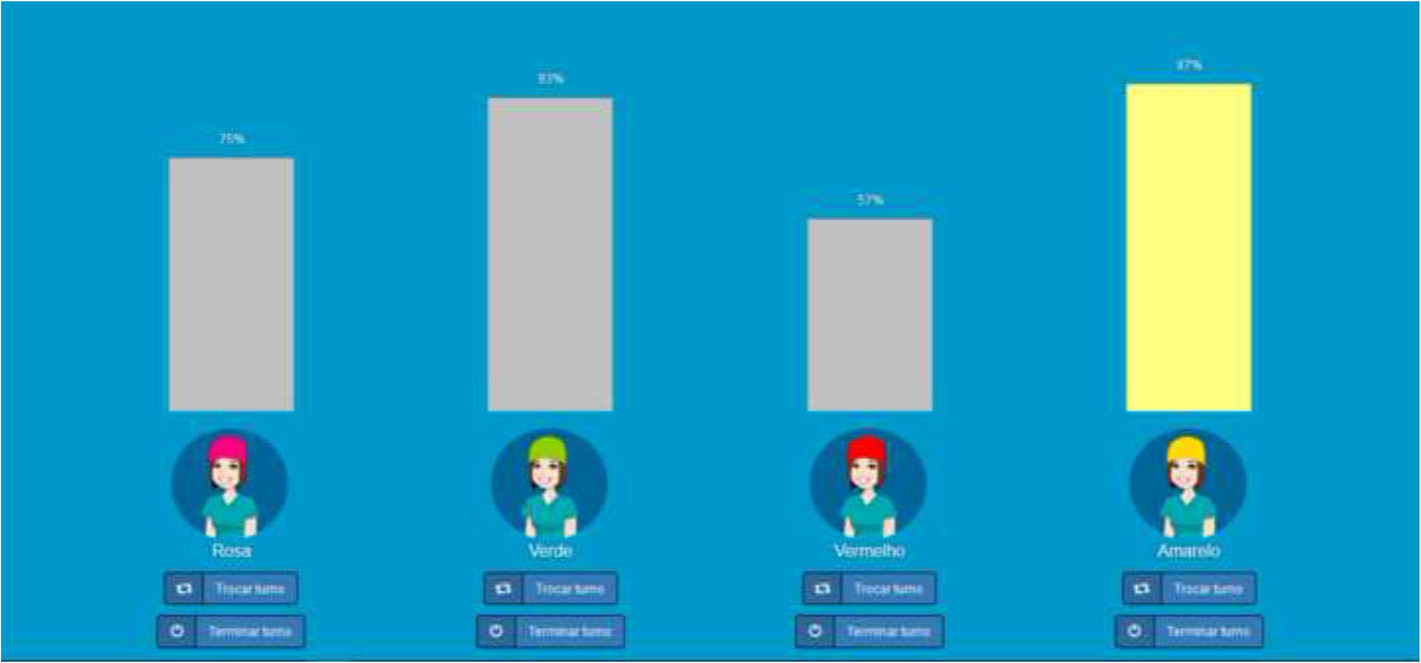
Dashboard component of the second instantiation of the gamification solution

The second instantiation of the dashboard screen is presented in Fig. 7. The avatars were changed to differ between each other in the cap colour, which corresponded to the name of the sensor they are using (pink, green, red or yellow — as the typical board game pieces).

A new component for the gamification solution was created. An e-mail functionality allowed HCWs to receive, after the end of their shift, a simple e-mail that provided feedback regarding their HH compliance rate and a link to a web application for further information. This application is to be explored outside HCWs labour hours, and makes use of several and distinct game components, to explore different mechanics and activate some dynamics.

**Fig. 8:**
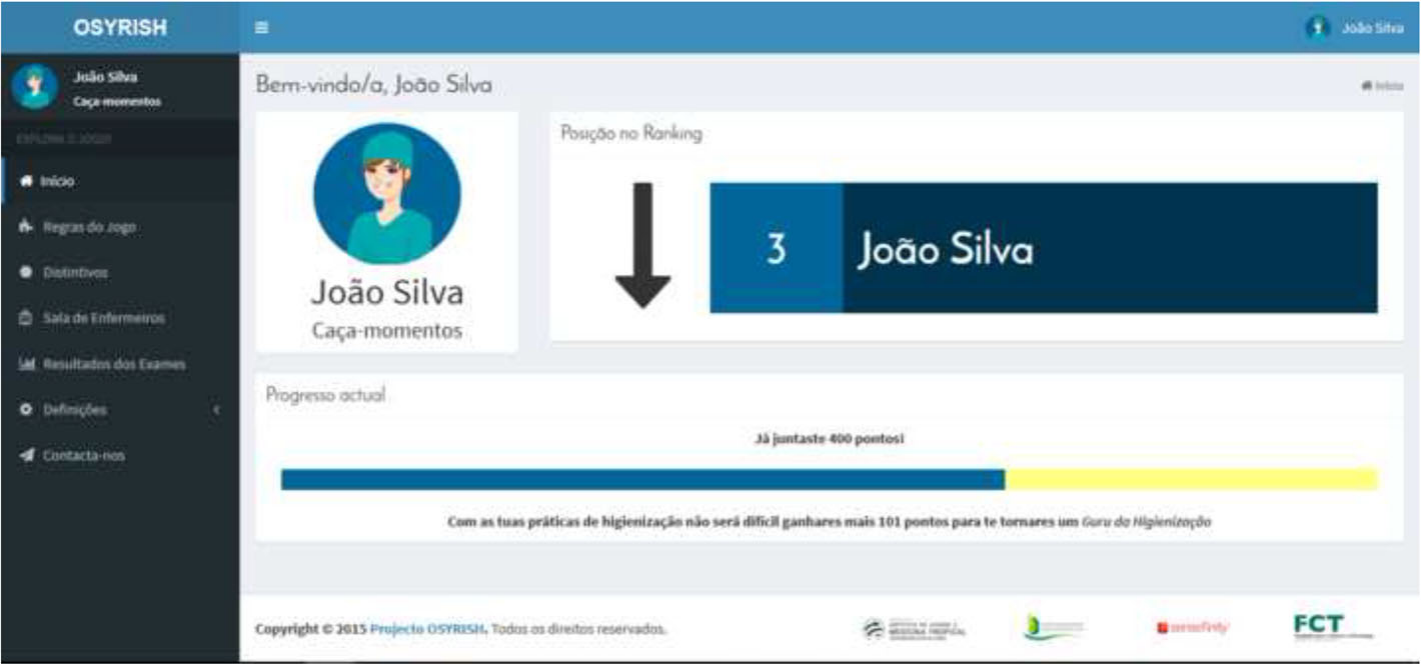
Homepage of the gamification component to explore outside labour hours. João Silva is a non-real name, used for training purposes only

After logging in, players were welcomed with some game information in the homepage (Fig. 8): their name, avatar, current level, their current score and how far they were from levelling up (combined with a progress bar, which provides visual feedback). Higher levels are more difficult to reach, meaning that you need more points to go from level 3 to level 4 than you need to go from level 2 to level 3.

A partial leaderboard presented the players’ current position in the ranking and whether they went up, down or maintained their position since the last leaderboard update. The researchers, following the conclusions of the focus group, decided to use a partial leaderboard instead of a complete one so that players with lower positions would not demotivate when realizing it was very hard to reach the top of the ranking, in an effort to keep the players engaged as long as possible.

Players are rewarded for a wide range of actions both in the ward (e.g., being the HCW with higher HH compliance rate in a shift) and in the game (e.g., sending suggestions to the research team) using badges. Categories are mostly organized by levels, which have an increasing difficulty level (e.g., for achieving level one you have to perform that action on one shift; level two requires you perform that action on ten shifts, and so on). Access to badges is limited to players that achieve a HH compliance rate above 70%, so that only HCWs with an acceptable performance are rewarded.

Besides acting as a reward, badges allow players to receive currency that can be used to acquire virtual goods on the virtual shop, which provides a wide range of assets players can “buy” to customize their game experience: elements to personalize their avatars, more stats information (like their score evolution over time), access to adjacent positions in the ranking, etc. With this, the goal is to provide HCWs with a sense of autonomy (which is proven to leverage motivation).

Currency differs from points in the sense that the last can only be achieved through their work in the ward and used to progress through levels. Nevertheless, since most badges are related to shift’s performance, players with higher scores are likely to achieve more badges, thus receiving currency, which depends on badges’ difficulty (more challenging badges are worth more currency).

A stats page allows players to generate graphs displaying relevant information, from which the only one available at the start (that means, HCWs do not have to acquire it from the shop) is HH compliance rate over a range of days or months (which can be defined by players) or during a shift (in which case the rate is presented by hours). This way, players can analyse their evolution and think of ways to improve their compliance (for example, if they realize they comply less when they receive a new patient in the unit, they can decide to be more aware at those times).

The social component of the solution is given by a page displaying all players, who can interact with each other and exhibit their status in the game, thus providing a sense of both competition and cooperation. A players’ small profile can include: name/username, level, description, number of HH compliances (since the game began), number of achieved badges and option of receiving messages. Nevertheless, they get to choose what information to display, being the minimum the name/username, level and avatar.

As players’ feedback is very important, a contact page was included, so that players can send suggestions, reviews or bug reports to the research team. Also, the main “game rules” are presented and detailed in the application, in case players have any doubt.

Regarding elements connection, points and levels are used to provide feedback; the partial leaderboard is used to stimulate competition; etc. Target dynamics are emotions, so that HCWs become aware and reflect about their performance, and relationships, which are related to cooperation and competition.

In this way, all of them are able to receive the feedback they are interested in with little additional workload (they only have to indicate they are starting and finishing a shift, and to consult their compliance rate during the shift) — which was one of the main concerns through the whole design and development phase. However, if they want to, they can follow the provided link and explore the application outside their labour hours. The goal with this is to induce some curiosity and see whether or not they are moved into using the solution.

To support the evaluation, Google Analytics (Google’s web analytics service to track and report website traffic [27]) was integrated with the IS to measure users interaction with the several gamification components. A small set of events (for example, players’ clicks on the links sent in the e-mail after their shifts) were tracked using mechanisms implemented in the IS itself.

Game elements used are summarized in Additional file 3: Appendix III.

### Simulation of the second instantiation and field studies on the hospital

This iteration also included a simulation after the instantiation completion and field studies, where the solution was studied in its real environment.

## Results

### Demonstration activity implementation

An Intensive Care Unit (ICU) of a Portuguese tertiary hospital was selected as the location to perform demonstration and evaluation activities during 10 months. This choice is justified by the fact that ICU wards are very demanding in terms of hygiene, and therefore a good place to perform a proof of concept as the one proposed in this research study. This ICU is composed by three rooms (one four-bed room and two two-bed rooms); each room is equipped with one sink, one ABHR dispenser near the entrance and one ABHR dispenser at the bottom of each bed.

To restrict the focus group, thus simplifying the research process, the decision to implement this instantiation only for nurses was made. Also, because they spend most of their time in the ward, it makes it easy for the research team to talk with them and gather feedback. From the 24 nurses working, six voluntarily participated in the research, being available to schedule three main focus groups with them, which occurred between 3 p.m. and 4 p.m (the period during when patients receive their families’ visit, where nurses are more available). There were four participants in each focus group, which were the ones assigned to the current nursing shift. Additionally, the chief nurse was always available to discuss some logistical questions.

Nurses were aged 25–60, meaning that this group crossed different generations. Even though this fact was considered, the research team decided to not to focus too much on this general characterization and developed the IS in collaboration with the nurses, and decided to infer their affinity with games based on their feedback after interacting with the system.

### Initial feedback from nurses

An initial focus group was conducted with nurses of the ICU with nothing but a concept and some design ideas, trying to understand their feelings about it.

Nurses enjoyed the concept and consider it is as a unique and good opportunity to receive feedback regarding their performance. Though they are sometime subject to audits, they said that this would give them a totally different and positive experience.

In the first focus group, they showed little interest in components like badges, virtual goods and content unlocking because it would require them to use the system outside their labour hours. However, they found the avatars experience funny and enjoyed the concept of leader-boards.

None of them expressed concerns regarding being monitored and having their identity exposed in the screen. They also believed that the language style used in the application should be more colloquial, because some people would not find adequate to use such system with a familiar language style.

### First iteration

After validating the concept with the nurses, a meeting with the chief nurse was performed so that the ward rooms’ configuration was presented and to decide where to place the beacons for the IPS.

### Preliminary tests

For the first preliminary experiment on Estimote® technology, the *Distance Demo App* was used to calculate beacons’ precision in estimating distances using a function called *computeAccuracy* (included in Estimote® Software Development Kit). This function returns a value corresponding to the estimated distance between a beacon and the device where the app is running. Furthermore, the correspondent signal strength values received from the beacons (Received Signal Strength Indication - RSSI) were also collected allowing for the calculation of distance.

Different scenarios were explored to measure the distance between beacons and a tablet running Android, using several beacons’ arrangements. Results showed that beacons were not always detected inside the promised range and, when they did, results were not accurate at all: the most accurate result still presented an error of 1,92 m.

**Fig. 9:**
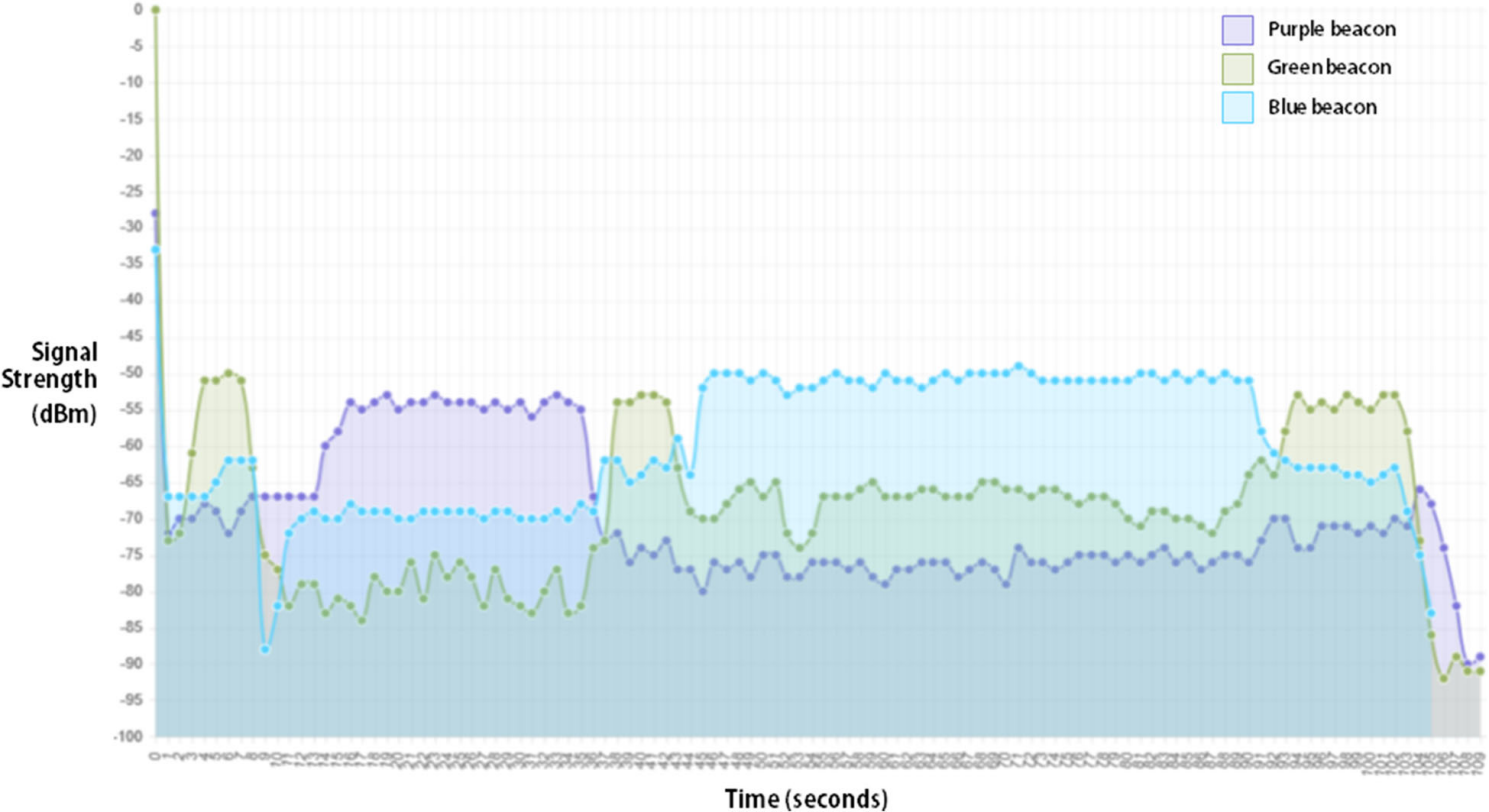
Graphic presenting proximity to Estimote ® beacons. Each line of different color represents a different beacon

After that, another different scenario was tested, using Estimote® Beacon’s main functionality: proximity detection. For that, a tool was developed to collect, store and present information regarding beacons’ RSSI values in the form of a graphic. A test was performed where beacons were placed a few meters apart and, carrying the tablet, the researcher would approach the beacons one at a time to check if the researcher’s path matched the graphic. The results are presented in Fig. 9. Each line of different colour represents a different beacon (green, purple, grey). Although times are not totally accurate, it is possible to identify in the graphic the sequence of movements performed (first the green line beacon, then the purple line beacon, etc.).

### Simulation of the first instantiation

Following these experiments, a simulation was conducted to test the whole solution. Three beacons were placed on a laboratory (corresponding to the entrance of a ward, a bed and an alcoholic-based hand-rub dispenser), the Android application installed on a tablet and a laptop placed where we could see the gamification screen. Data was well processed and presented in the screen, but there were some problems in detecting beacons. Sometimes the tablet was placed right near a beacon and the application did not detect it, while other times (in the exactly same situation) its presence was detected. Also, the signal was much more unstable than when beacons were purchased and battery levels fell down very quickly.

### Second iteration

Having collected feedback from focus groups during first iteration, especially in the final one, the instantiation was deeply improved. Two examples of how this feedback was implemented are the e-mail functionality, created to cope with nurses’ sceptical attitude towards using game elements that required them to access the solution outside their work time, and the decision to include the logic to start and finish a shift in the dashboard screen.

In a new focus group following instantiation completion, nurses approved the improvements, both in terms of design and functionality, but warned that they would only be able to make suggestions and assess functionalities’ usefulness by the time they start using the application on a regular basis.

### First field study on the hospital

Simultaneously, the IPS solution was designed in collaboration with Sensefinity® and, when everything was completed, a field study was scheduled in a meeting with the chief nurse. A total of 26 beacons were deployed in all the desired positions of ICU’s three rooms. One screen was placed in the nurses’ room, connected to one of ICU’s computers, presenting the dashboard component of the gamification solution to provide real-time feedback regarding their HH compliance. During this 5 days’ study, only the IPS was tested. It was noticed that the GSM coverage in the hospital is very weak, except for one specific Portuguese operator, which Sensefinity informed to be the one associated with the telecommunication cards used in the system. Sensefinity®’s solution presented several hardware problems, which were solved by their collaborators as they were spotted. However, in the end the system was sending more messages than expected (without a visible pattern) and, for that reason, all the equipment was removed from the ICU for Sensefinity® to perform further refinements. Foremost, the research team did not have access to the hospital Wi-Fi network, due to hospital’s restrictions, which was an obstacle to refine the IS that was hosted on the cloud in real-time.

Nurses did not have a chance to experience the solution during this period, but it was possible to collect more feedback from them. They indicated that beacons placed near the beds often fall from their places, especially when beds were cleaned. One case where a beacon had fallen during a surgery was also reported.

### Simulation of the second instantiation

Four months later, Sensefinity® returned the equipment and some validation tests were performed. Results showed that, regardless of Sensefinity®’s intervention on the equipment, the system was still very unstable and sending far more messages than those expected. Sensefinity® assumed this instability as a technological limitation the research team needed to comply with. Following this, sensors’ outputs were analysed to discover relevant patterns that could be used to filter data and improve IPS’s accuracy. However, these efforts were unsuccessful.

### Second field study on the hospital

**Fig. 10:**
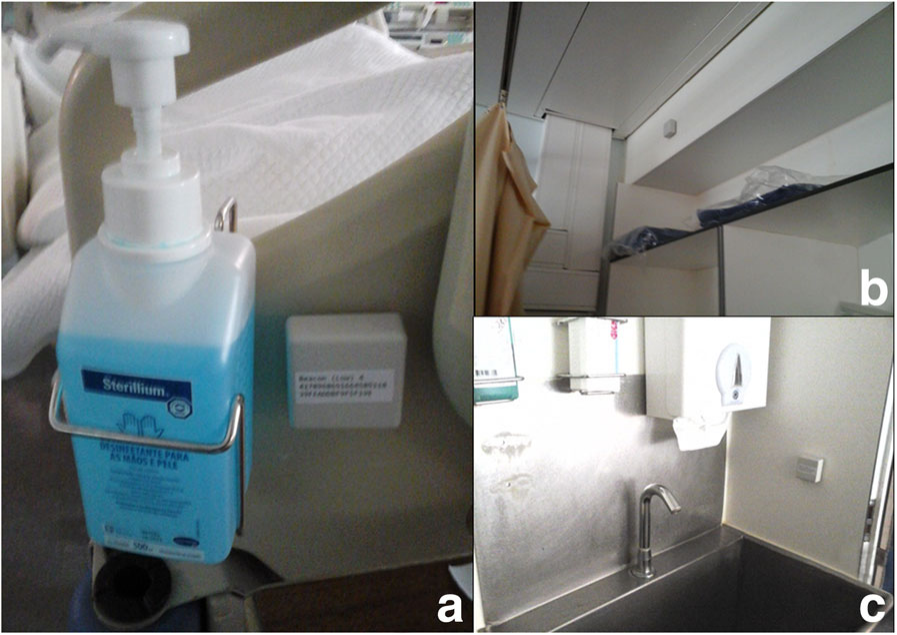
Beacons placed in the ICU ward. **a** Near an ABHR dispenser placed on a bed; **b** Near a bed; **c** Near a sink

Despite this, a second field study was scheduled in a meeting with the chief nurse and subsequently performed, where 12 beacons were deployed on only two rooms of the ICU (one two-bed and one four-bed rooms) due to some restrictions Sensefinity® had concerning the available equipment. In Fig. 10 one can find examples of beacons placed in the areas of interest. Because in the four-bed room ABHR dispensers are not usually fixed at the bottom of the beds (instead, they are fixed in a table, which in positioned near the bottom of beds), the chief nurse changed their position and urged all the nurses not to change it back.

A test to analyse IPS behaviour was conducted, where a nurse was handed a tag for her to carry during 2 hours. During this time, the nurse was mostly in one room (approximately 1 h and 45 min), and after that went to the nurses’ room (where the IPS was not installed). Only three location messages were received, being one of them from a beacon placed in a room the nurse never entered. When confronted with these results, Sensefinity® stated that this might be due to the fact that the system used telecommunication cards from a different operator than the one with acceptable coverage, contradicting what was first told. These messages were not sufficient to generate information to be displayed in the dashboard component of the gamification solution. Nurse’s feedback was that she barely noticed the location tag she carried on her pocket, which did not affect her practices.

Another important thing to highlight was that, few days after deploying the system, ABHR dispensers placed in the beds of the four-bed room were placed back on the tables, despite chief nurse’s advice for the nurses to not to change them back.

### Overview of indoor location technologies

Despite not being the initial main focus of this research, the search for an accurate IPS constituted an important part of this project. Table 2 presents a summary of both IPS’s features, strengths and weaknesses.

**Table 2:** Summary of Estimote® and Sensefinity® main features, strengths and weaknesses

	Estimote®	Sensefinity®
Features	• Wireless beacons; • Based on BLE technology; • Signals broadcasted are detected by applications installed on mobile devices; • Main functionality is proximity detection; • The app needs Wi-Fi or GSM to communicate over the Internet; • Software Development Kit and example Demo app are provided.	• Wireless beacons and tags; • Based on BLE technology and a proprietary protocol operating on the 2.4GHz frequency band; • Provides a variety of functionality (proximity, direct location, etc.); • Communicates over the Internet using gateways via GSM; • Webservice provided to extract the data.
Strengths	• Not very expensive; • Accurate when providing proximity.	• Not very expensive; • Provides all necessary equipment to build an IPS; • No need for an already existent network;
Weaknesses	• Need to buy other equipment, namely mobile devices; • Demands the existence of Wi-Fi network or to use GSM; • Not good for estimating direct distances; • Very susceptible to interferences; • Batteries levels decrease very fast.	• Demands the existence of GSM coverage; • Hardware is very vulnerable to damage; • Very susceptible to interferences; • Beacons fall off frequently from their places.

It is possible that some Estimote® characteristics have not been discussed here since this solution was never deployed in the ICU. For example, probably these beacons would also fall from their places.

## Discussion

As far as it is known, presented results constitute a pioneer initiative to assist HAIs problem. Although there are several successful examples of gamification systems in health contexts, the majority of them are related to personal healthiness (especially fitness apps) and require mobile devices usage [28]. Little evidence was found of solutions aiming at improving HCWs practices, where mobile devices’ usage is against the standard rules [29].

Nurses approved this measure as an opportunity to improve their performance, which confirms the initial results. Although they stated they were only interested in knowing their HH compliance rate and were not willing to use the gamification application, the research team continued to invest in this approach. It was considered that nurses’ opinion, which was based on general concepts and drafts, could change after seeing a developed instantiation and consider the chance of using it on a daily basis, which was the case. If nurses do engage with gamification approaches and if this engagement leads to a performance optimization, knowing that a decrease in HH compliance rate lead to an increase in the number of patients affected by HAI [3], maybe the symmetric effect can happen.

Consideration of human factors to promote a new and better habit was not only present on mechanics like the real-time feedback displayed in a fun and engaging way. By involving nurses since the beginning and taking their feedback into consideration during the whole process, nurses developed a sense of trust in the research team (thus they were more willing to accept being monitored) and it was possible to align the solution with their needs. A good example is the e-mail functionality already presented and justified.

To engage and motivate nurses in achieving the goal of better HH practices, an instantiation was developed based on two auxiliary models. The power of games was used to leverage HCWs motivation, but this cannot be done without the location information that allows the application of the “My five moments for hand hygiene” framework. For this information to be useful, a good choice must be done regarding the architecture of the IPS underlying it, selecting the technology(ies), method(s) and technique(s) that better fit the requirements. Since there is no standard technology for indoor location (like we have the Global Positioning System (GPS) for outdoor location), due to indoor environments’ higher complexity [30], this is not a trivial task.

Two innovative and commercial IPS solutions were considered and tested.

Tests performed on Estimote® beacons showed that estimating distances to them is unfeasible for the present work, since results were not accurate at all, but with a proximity-based technique it is possible, at least, to describe one’s movements. The simulation allowed to conceptually validate the IS, since it was possible to detect movements using proximity and to quantify compliance with some precision. However, it was observed that beacons did not present a stable behaviour, which can be due to the fast decrease in batteries’ level. The research team decided that no advantages would arise in performing a field study using this technology, since results were not positive and a hospital environment poses much more interferences that affect beacons’ performance. Despite the reason underlying beacons’ weak performance, a fact is that the numbers promised by Estimote® regarding beacons’ range and accuracy are far away from reality.

Although Sensefinity®’s solution was deployed, it was not possible to fully test it with the gamification because several problems were faced during the iteration. GSM weak coverage on the hospital can be one of the problems affecting IPS’s correct functioning, besides not allowing the research team to use an internet hotspot to solve the previously stated problem. This was also a major problem, since it made it difficult to the team to analyse results and solve problems in real-time. This, and the constantly malfunctioning hardware, are two of the reasons that might led to signal’s instability, which Sensefinity® pointed out as a technological limitation that the research team needed to comply with. Regardless of research team’s efforts, it was evident that Sensefinity®’s solution still needs considerable and deep refinements before it can work properly and be applied in a healthcare context.

Other major consideration is the fact that beacons are often falling from their places, since it does interfere not only with the system correct functioning, but also with nurses’ regular practices. A less invasive IPS should be used, since beacons placed in the nurses’ field of action can interfere with their regular practices (by falling off frequently or by implying a change of configuration in the ward).

Due to all these limitations, it was not possible to perform the planned long-term tests with the automated monitoring system, with and without gamification. This also means that it was not possible to quantitatively assess gamification impact and answer one of this research’s main question: can gamification change HCWs behaviours regarding HH practices?

**Fig. 11:**
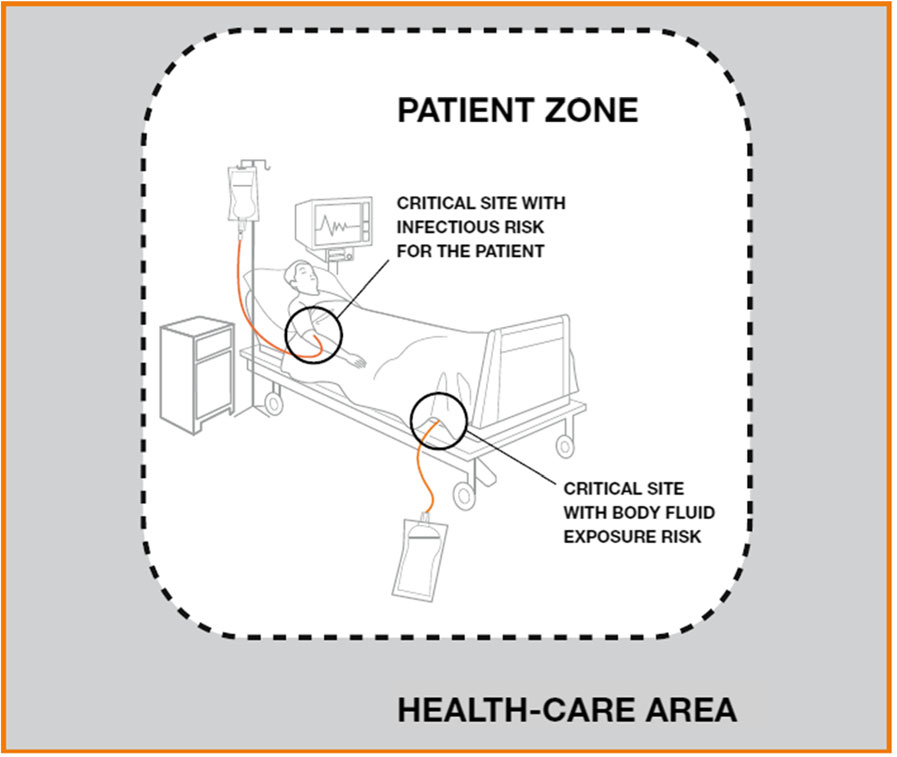
Unified visuals for “My five moments for hang hygiene” (taken from [4])

This system suffers from some of the typical limitations associated with automated monitoring systems. It is only able to detect compliance with moments 1, 4 and 5 of WHO’s “My five moments for hand hygiene” framework (Fig.11). The IS can detect moments before touching a patient, after touching a patient and after touching patient surroundings (meaning that the HCW had contact with the patient zone but did not touch the patient) because these imply a significant change of location, from the general healthcare area to a specific patient zone, and vice versa. Because moments before performing a clean/aseptic procedure (such as handling an invasive device) and after exposure risk to body fluids (like excretions) happen inside a patient zone, the IS does not detect its occurrence, since there is no change of location. According to several studies, moments 1, 4 and 5 account for 80% of the total HH opportunities [8]. There is some potential to observation bias: although there is no presence of a physical observer, nurses may have the sense of being observed, which can lead to higher (thus, unreal) HH compliance rates [4].

It is important to discuss which and how other hospital initiatives regarding HH could have influenced the outputs of the gamification intervention. In the ICU where the research was conducted, informative posters about HH technique were placed near all sinks, which could act as reminders for HCWs. Additionally, professionals working in that service were regularly trained on “My five moments for hand hygiene” framework and the service was periodically subject to external and internal audits on the same subject. Even though such initiatives were expectable to highly influence HH compliance, nurses stated during focus groups that they do not felt comfortable during audits and do not believed its outputs reflected the truth regarding HH performance (observation bias could have had a great impact here). Additionally, these results are not individualized and are disseminated large months after audits occurred. These assumptions are confirmed by a previous study conducted in the ICU where two nurses were observed during an 8 hours shift (8 a.m. – 5 p.m. — with 1 hour lunch). The overall HH compliance rate of observed HCWs was 69,8% [95% CI: 67.0–72.5%], which is in line with findings from the most recent report on hand hygiene published by the Directorate-General of Health (DGS) and in line with international studies [31, 32]. Since HH performance is expected to be as high as possible, it is easy to understand that there is much room to improvements.

Although indoor location technologies strongly evolved during the last few years and have been successfully applied in areas like retail, their precision is still not enough to be applied in the healthcare field, where the areas of interest are much smaller (down to centimetres). Subsequently to this research, another technology, inBeacon® [33], was subject to some preliminary tests in conjunction with three RadBeacon Dot from Radius Networks [34]. This platform, which allows the creation of context awareness programs using beacon technology, presented slightly better results than the other two technologies, but they are still not sufficient to cope with this use case.

Capabilities of automated monitoring systems, based on location information, can be augmented by combination with other methods, such as electronic counting devices for ABHR consumption. These devices can detect HH actions by recording accesses to a ABHR dispenser, but are not able to detect HH opportunities, thus cannot calculate HH compliance rate. Alone, these devices are also not able to distinguish who performed the HH action [8]. However, these limitations can be surpassed when crossed with location information: if an ABHR was accessed, the automated monitoring system would detect which professional was near it and could associate the action with him or her.

In further trials, it would be of importance to include physicians and other professionals. Although wards share many characteristics they have often different organizations and cultures and follow different care models. To gather a richer set of results, the system should be deployed in other wards to be studied.

This research presented an example of one digital transformation in healthcare services. It is important to reflect on this reality, which seems to be much easier that it actually is. At the starting point of this project, the research team believed that it would not take long to develop or acquire an IPS to integrate with our solution. However, because the required precision is much higher than the one current IPS technologies offer, this was a much slower process.

Another concern is related to HCWs practices’ monitoring, which brings up privacy issues. There is a danger that management will try to use this sort of information against the workers, which in turn will make them less willing to use the system. One might argue that management can make the system’s usage obligatory. By making the “game” mandatory, one is negatively impacting the effects of gamification, since games are, by definition, voluntary [35]. It is important to have all these considerations in mind when developing a technological solution to a non-technological practice, where not only technological, but especially management, organizational and human issues can and will arise.

## Conclusions

The impact of gamification on HH compliance is still under evaluation. Even though it was not possible to fully deploy the solution in the ICU, so far the results show that the IS is promising in improving nurses’ awareness.

By involving nurses in the research since the beginning, it was possible to align the IS with their needs, which was confirmed by the results and their feedback. In projects like these, where a solution is being designed for the benefit of someone, is important to involve them since the very beginning in order to enable a higher sense of ownership in the process and better understand end users’ requirements.

It is also important to mention that there is a strong need to test and improve indoor location technologies, since their precision is far from ideal to be applied in the healthcare field.

DSRM was a valuable methodology that allowed the study of the co-design of the system along with the sense of ownership by the nurses’ participating in the study.

This study was a first step towards the implementation of an innovative approach to support nurses HH performance in a more active and friendly way. Finding an accurate IPS to track HCWs inside the hospital is a mandatory task regarding future work. Further tests might include involving other HCWs (such as physicians) and deploy the IS in units with care models different from the ICU. To finish, a key concern is how to keep HCWs engaged when the novelty feeling disappear. This could be done, for example, by planning new gamification feature releases.

## Additonal files

Additional file 1: Appendix I. WHO’s “My five moments for hand hygiene” framework. Detailed description of WHO’s “My five moments for hand hygiene” framework, which links specific moments to HH opportunities and was applied in this study. (DOCX 17 kb)
Additional file 2: Appendix II. Game Elements. Presentation and discussion of the list of game elements proposed by Werbach and Hunter, which was used as a basis to build the instantiation’s gamification component. (DOCX 17 kb)
Additional file 3: Appendix III. Game Elements applied in the solution. List of the game elements applied in both instantiations, categorized accordingly to Werbach and Hunter’s list, and to the part of the gamification solution they appear in. (DOCX 12 kb)

## Abbreviations

ABHR: Alcohol-based hand-rub
BLE: Bluetooth Low Energy
DSRM: Design Science Research Methodology
GPS: Global Positioning System
GSM: Global System for Mobile Communications
HAI: Hospital acquired infections
HCW: Healthcare workers
HH: Hand-hygiene
ICU: Intensive Care Unit
IPS: Indoor positioning system
IS: Information System
RSSI: Received Signal Strength Indicator
WHO: World Health Organization
