# Supplementary material for: How can information systems provide support to nurses’ hand hygiene performance? Using gamification and indoor location to improve hand hygiene awareness and reduce hospital infections
Source: BMC Med Inform Decis Mak. 2017 Jan 31;17:15. doi: 10.1186/s12911-017-0410-z (PMC5282776; doi:10.1186/s12911-017-0410-z)
Supplement: Additional file 1: Appendix I. — WHO’s “My five moments for hand hygiene” framework. Detailed description of WHO’s “My five moments for hand hygiene” framework, which links specific moments to HH opportunities and was applied in this study. (DOCX 17 kb) [file 12911_2017_410_MOESM1_ESM.docx]

# **Appendix I: WHO’s “My five moments for hand hygiene” framework**

“My five moments for hand hygiene” is a framework proposed by WHO for understanding, training, observing and communicating HH performance [4]. It links specific moments to HH opportunities. To help identifying those moments, a geographical representation (focused on a single patient) was created and the five moments correlate to them (Figure 11).

Patient zone contains a patient X and his/her immediate surroundings and health-care area contains all other surfaces (other patients and health-care facilities). Within patient zones we have critical sites with infectious risk for the patient (body sites or medical devices that have to be protected against microorganisms), critical sites with body fluid exposure risk (body sites or medical devices that potentially lead to hand exposure to body fluids and bloodborne pathogens) and critical sites with combined risk (include risks from previous critical sites).

The moments proposed in the framework where HH must be performed are:

1. **Before touching a patient**

Period between the last contact with the health-care area and the first contact with the patient zone;

1. **Before a clean/aseptic procedure**

Period between the last exposure with any surface (either from health-care area or patient zone) and the access to a critical site with infectious risk for the patient or critical site with combined infection for patient care (this includes handling an invasive device - regardless of whether gloves are used or not - and moving from a contaminated body site to a clean body site of the patient);

1. **After body fluid exposure risk**

Period between performing a care task associated with a critical site with body fluid exposure risk for the patient or critical site with combined infection (for example, when contacting with body fluids or excretions, mucous membranes, non-intact skin or wound dressings; after removing gloves; if moving from a contaminated body site to a clean body site if the patient) and the first contact with any other surface.

1. **After touching a patient**

Period between the last contact with the patient zone where the HCW touched the patient and the first contact with any surface in the health-care area;

1. **After touching patient surroundings**

Period between the last contact with the patient zone (with inanimate objects – including medical devices – in the immediate vicinity of the patient) without touching the patient and the first contact with any surface in the health-care area.

Examples of care situations for each moment are presented in Table 3. As we can see, some moments occur at the same situations (for example, oral/dental care create an opportunity for the moments 2 and 3) or sequentially (for example, before contact with one patient might happen right after contact with another patient). In such cases, HCWs only need to perform HH once.

Table 3 - Examples of care situations when each of the five moments occur (adapted from [4])

| **Moment** | **Examples of care situations when the moment occurs** |
| --- | --- |
|  |  |
| 1 – Before touching a patient | Shaking hands; helping a patient to move around; getting washed; taking pulse; chest auscultation; abdominal palpation |
| 2 – Before clean/  aseptic procedure | Oral/dental care; secretion aspiration; skin lesion care; wound dressing; subcutaneous injection; catheter insertion; food, medication and dressing sets preparation. |
| 3 – After body fluid exposure risk | Oral/dental care; secretion aspiration; skin lesion care; wound dressing; subcutaneous injection; drawing and manipulating any fluid sample; clearing up urines, faces, vomit; handling waste; cleaning of contaminated and visibly soiled materials or areas. |
| 4 – After touching a patient | Shaking hands; helping a patient to move around; getting washed; taking pulse; chest auscultation; abdominal palpation |
| 5 - After touching patient surroundings | Changing bed linen; holding a bed rail; cleaning bedside table. |
