# Supplementary material for: How can information systems provide support to nurses’ hand hygiene performance? Using gamification and indoor location to improve hand hygiene awareness and reduce hospital infections
Source: BMC Med Inform Decis Mak. 2017 Jan 31;17:15. doi: 10.1186/s12911-017-0410-z (PMC5282776; doi:10.1186/s12911-017-0410-z)
Supplement: Additional file 2: Appendix II. — Game Elements. Presentation and discussion of the list of game elements proposed by Werbach and Hunter, which was used as a basis to build the instantiation’s gamification component. (DOCX 17 kb) [file 12911_2017_410_MOESM2_ESM.docx]

# **Appendix II: Game Elements**

The list of game elements proposed by Werbach and Hunter [16] is divided into three categories:

- **Dynamics:** elements at a higher level of abstraction; they correspond to the overall view of the gamified system that have to be carefully considered and managed, but are not directly entered in the game.
- **Mechanics:** processes that drive the action forward and create engagement. Each mechanic can be used to achieve one or more dynamics.
- **Components:** concrete forms that dynamics and mechanics can take (one component might be connected to one or more dynamics or to one or more mechanics).

In Table 4 we present some examples of game elements, following this classification. we present some examples of game elements, following this classification.

**Table 4** – Some game elements, classified according to the framework proposed by [16][16]

| **Classification** | **Concept** | **Description** |
| --- | --- | --- |
| **Dynamics** | Emotions | Curiosity, competitiveness, frustration, happiness |
|  | Relationships | Social interactions |
| **Mechanics** | Competition | One player or group winds, and the other loses |
|  | Cooperation | Players must work together towards a shared goal |
|  | Feedback | Information about how the player is doing |
|  | Win States | Objectives that makes one player or group the winner – draw and loss states are relative concepts |
| **Components** | Avatars | Visual representations of a player’s character |
|  | Badges | Visual representations of achievements |
|  | Levels | Defined steps in player progression |
|  | Points | Numerical representations of game progression |

As an example of how these elements can be interlinked between categories, consider the Kinect Sports® game. It is composed by a set of sports simulation and mini games, and by getting out of the sofa people feel several **emotions** (which depend on the individual: one might feel happy, while others might sense frustration, etc.) and, if playing with other people (which is the common scenario), they are also developing **relationships**.

Emotion dynamic can be mapped to a sense of **competition** or **cooperation**, or be evident during **challenges** and while receiving instantaneous **feedback**. Playing by **turns** is connected to the relationship dynamic. At the beginning of the game, players chose their **avatar** and can be organized in **teams** (boosting both a competition and cooperation feeling, related to both of this game’s main dynamics). Games can consist of direct **combats** (which implies a relation with other players), where players award **points** and can achieve a **win state** (a mechanic that usually triggers positive emotions).

Despite the fact that not all game elements are tangible, they are all of importance for defining and understanding the game.

Furthermore, one must understand that the goal is not to incorporate all these elements in a solution; instead the ones that better serve the purpose shall be picked after defining variables like goals, desired target behaviours and players.
