# Supplementary material for: How can information systems provide support to nurses’ hand hygiene performance? Using gamification and indoor location to improve hand hygiene awareness and reduce hospital infections
Source: BMC Med Inform Decis Mak. 2017 Jan 31;17:15. doi: 10.1186/s12911-017-0410-z (PMC5282776; doi:10.1186/s12911-017-0410-z)
Supplement: Additional file 3: Appendix III. — Game Elements applied in the solution. List of the game elements applied in both instantiations, categorized accordingly to Werbach and Hunter’s list, and to the part of the gamification solution they appear in. (DOCX 12 kb) [file 12911_2017_410_MOESM3_ESM.docx]

# **Appendix III: Game Elements applied in the solution**

**Table 5 –** Game elements used in the gamification solution

|  | **Dashboard** | **Player Application** |
| --- | --- | --- |
| **Components** | Points | Avatars  Badges  Leaderboard (partial)  Points  Levels  Virtual Goods |
| **Mechanics** | Competition  Feedback  Win State | Competition  Cooperation  Feedback  Resource Acquisition |
| **Dynamics** | Emotions  Relationships | |
